# Relationship Between Sleep‐Related Worry, Compassion Fatigue, and Retention Intention Among Nurses: A Multicenter Cross‐Sectional Study

**DOI:** 10.1155/jonm/7964982

**Published:** 2026-06-26

**Authors:** Feng Peng, Xiaoying Zeng, Jue Wu

**Affiliations:** ^1^ Department of Operating Room, The First Affiliated Hospital of Chengdu Medical College, Chengdu, Sichuan, China, scu.edu.cn; ^2^ West China Hospital of Sichuan University-Ziyang Hospital, Ziyang Central Hospital, Ziyang, Sichuan, China, scu.edu.cn; ^3^ Science and Education Department, Sichuan Taikang Hospital, Chengdu, Sichuan, China, scu.edu.cn

**Keywords:** compassion fatigue, nurse, retention intention, sleep-related worry

## Abstract

**Aims:**

To examine the current situation of sleep‐related worry, compassion fatigue, and retention intention among nurses in general hospitals in China, and explore whether there might be any connection among the three.

**Design:**

This study adopts a cross‐sectional and descriptive design.

**Methods:**

From January to February 2024, a survey was conducted on 1831 on‐duty nurses from eight tertiary general hospitals in China (Sichuan, Hubei, and Shenzhen) using a convenience sampling method. The survey instruments included general information questionnaires, The Anxiety and Preoccupation about Sleep Questionnaire, The Compassion Fatigue Short Scale, and The Questionnaire for Nurse Intention to Remain Employed. Collected data were subsequently analyzed.

**Results:**

The mean scores for sleep‐related worry, compassion fatigue, and retention intention among nurses in general hospitals in China were 30.40 ± 11.28, 48.03 ± 27.26, and 22.79 ± 3.71, respectively. Significant negative correlations were observed between retention intention and both compassion fatigue and sleep‐related worry, while a significant positive correlation was found between sleep‐related worry and compassion fatigue. Furthermore, compassion fatigue partially mediated the relationship between sleep‐related worry and retention intention, with the mediation effect accounting for 65.15% of the total effect.

**Conclusion:**

This study indicates that nurses’ sleep‐related worry and compassion fatigue may be related to their retention intention.

**Implications for Nursing Management:**

This study reveals that there may be a certain connection between nurses’ sleep‐related worry, compassion fatigue, and retention intention. It is suggested that nursing managers should prioritize improving nurses’ sleep quality, alleviating their sleep‐related worry, and enhancing organizational support. These measures may to some extent alleviate the phenomenon of compassion fatigue and even change the retention intention of nurses, thereby effectively curbing the brain drain of talents.

## 1. Introduction

Nurses constitute an essential element of the healthcare system and function as a fundamental pillar in maintaining the effective operation of medical services. Their occupational well‐being and workforce stability are directly and substantially associated with the quality and safety outcomes of patient care delivery [1]. However, the global healthcare sector is currently facing significant challenges, characterized by a dual predicament of nurse shortages and high turnover rates. This crisis not only threatens the sustainability of healthcare systems but also has profound and far‐reaching implications for the quality of patient care [2–4]. With the accelerating aging of the population, the growing burden of chronic diseases, and the escalating demand for higher health services, the imbalance between the supply and demand of nursing resources has become increasingly pronounced [5]. Against this backdrop, enhancing nurses’ retention intention and consistently advancing the development of the nursing workforce have become critical issues that require in‐depth research and effective resolution in the field of healthcare management today. Research indicates that the intention to stay, a critical antecedent in predicting actual turnover behavior, is influenced by multiple factors across different levels, with an individual’s physical and mental health playing a particularly significant role [6]. Among the numerous health risks, sleep worry and compassion fatigue are particularly prominent [7, 8]

Sleep‐related worry is characterized by persistent concern about the adverse effects of insufficient or poor‐quality sleep [9]. The Conservation of Resources (COR) Theory suggests that individuals are inclined to actively acquire, maintain, develop, and protect their valued resources, including energy, positive emotions, and cognitive abilities. When these resources are lost or perceived to be at risk, individuals may experience significant psychological stress [10]. The sleep disturbances experienced by nurses are not solely attributable to disruptions in circadian rhythms but rather originate from systemic resource depletion resulting from the interplay between occupational demands and organizational environments. Given that nursing involves continuous shift work and high‐intensity responsibilities, practitioners are inherently vulnerable to sustained resource exhaustion, which predisposes them to tension, stress reactions, job burnout, and other adverse emotional outcomes [11]. If sleep worry persists, it is highly probable that resource consumption will be further intensified. Relevant studies have demonstrated that worry can activate the central nervous system by increasing emotional distress, thereby partially impeding the normal onset of sleep [12]. This contributes to a vicious cycle of “worry–insomnia–increased worry,” which ultimately results in inadequate physiological recovery of energy. Meanwhile, concerns about sleep loss and the persistent rumination on potential adverse consequences also deplete significant cognitive resources (e.g., attention) and positive emotional resources (e.g., resilience) [12]. Research indicates that nurses experiencing poor sleep quality are more susceptible to emotional exhaustion [13]. This prolonged and chronic state of resource depletion may lead nurses to perceive their work environment as characterized by “high threat and low reward.” According to the COR theory, when individuals perceive a significant depletion of their resources and encounter difficulties in replenishing them, the most immediate coping strategy aimed at preserving remaining resources is to withdraw from the current resource‐intensive environment. This response is typically reflected in reduced organizational retention intentions and, in more severe cases, may culminate in actual turnover behavior [14]. Based on the aforementioned analysis, this study formulates Hypothesis 1: nurses’ sleep‐related worry may be somehow linked to compassion fatigue and retention intention.

Compassion fatigue refers to a state of physical, psychological, social, and spiritual exhaustion experienced by professionals in helping fields. It arises as a result of prolonged and intense exposure to the traumatic experiences of those they assist, coupled with excessive emotional involvement in their lives. This condition is primarily characterized by diminished compassion and professional satisfaction, feelings of emotional numbness or detachment from work, and, in some cases, symptoms of vicarious trauma [15, 16]. From the perspective of resource conservation theory, compassion fatigue essentially stems from the continuous depletion of emotional resources. As medical professionals who maintain the closest contact with patients, nurses are required by the nature of their work to consistently provide deep emotional engagement and care, which inevitably places them at a higher risk of experiencing compassion fatigue [16]. In the daily work of nurses, when excessive emotional labor is required and the consumption of emotional resources surpasses an individual’s capacity for recovery, the phenomenon of “compassion fatigue” may occur. Numerous studies have demonstrated that compassion fatigue exerts a predictive influence on nurses’ intention to remain in their roles [17], a mechanism consistent with the “resource investment principle” outlined in the COR theory. Specifically, when individuals anticipate that their resource investments will not yield commensurate returns, they may choose to cease further resource expenditure in order to prevent additional depletion [10]. Therefore, when a nurse’s emotional resources are nearing depletion and insufficient external support is available, resignation may serve as an adaptive strategy to prevent further personal resource drain. Building on this reasoning, this study formulates Hypothesis 2: the nurses’ compassion fatigue might be related to their retention intention.

The link between sleep‐related worry and compassion fatigue can be attributed to the reciprocal depletion of both physical and psychological resources. According to the COR theory, a “resource rut effect” exists among various types of resources, indicating that the depletion of one resource can trigger a cascading loss of other related resources [10]. For the nursing group, the shift work system is the most direct factor influencing sleep patterns. Research indicates that nurses working 8‐h shifts experience significantly lower levels of compassion fatigue compared to those working 12‐h shifts, and the duration of vacation time is negatively correlated with the level of compassion fatigue [13]. This suggests that the time spent away from the work environment constitutes a critical period for the restoration of physical and mental resources. However, the shift system significantly reduces the opportunity for restorative sleep, thereby placing nurses in a state of chronic sleep deprivation. Sleep, as a fundamental source of energy, directly diminishes an individual’s capacity for emotional regulation when it is insufficient [13]. Long‐term sleep deprivation is likely to exacerbate compassion fatigue among nurses, which manifests as a persistent emotional burden. The sustained depletion of personal resources may lead nurses to reassess the value of their current positions, ultimately influencing their decision to remain in their roles. Based on the aforementioned rationale, this study proposes Hypothesis 3: compassion fatigue may regulate the relationship between nurses’ sleep‐related worry and their retention intention.

However, current research on the relationship between nurses’ mental health and career stability still has significant limitations. On the one hand, most studies focus on the independent effects of sleep‐related worry or compassion fatigue and rarely incorporate both into the same framework to explore their interaction; on the other hand, empirical studies on the Chinese nursing population are particularly scarce, especially regarding the mechanism of the interaction among sleep‐related worry, compassion fatigue, and retention intention. Compassion fatigue, as the core manifestation of nurses’ long‐term emotional exhaustion, whether it plays a key mediating role between sleep‐related worry and retention intention, currently lacks systematic empirical evidence. Therefore, this study aims to focus on examining the potential mediating role of compassion fatigue in the relationship between sleep‐related worry and retention intention in the Chinese nursing population. A thorough understanding of this dynamic mechanism and the subsequent development of targeted intervention strategies based on this understanding not only holds significant value at the theoretical level but also has profound implications in the practical field. This helps to alleviate the psychological burden of nurses, reduce the staff turnover rate, ensure the stability of the nursing team, and ultimately improve the overall quality of medical services.

## 2. Methods

### 2.1. Design

This study employed a cross‐sectional design. The data were collected between January and February 2024. A total of 1840 questionnaires were distributed and 1831 were returned (a recovery rate of 99.51%). The Strengthening the Reporting of Observational Studies in Epidemiology (STROBE) guidelines were followed.

### 2.2. Participants

From January to February 2024, a survey was conducted on 1831 on‐duty nurses from eight tertiary general hospitals in China (Sichuan, Hubei, and Shenzhen) using a convenient sampling method. The inclusion criteria were as follows: (1) Registered nurses currently serving in active roles; (2) employment duration of 3 months or longer; and (3) willingness to voluntarily participate in the study. Exclusion criteria were as follows: (1) Nurses who are on leave or attending external training programs outside the hospital; (2) nurses who were absent during the survey period; and (3) nurses who do not participate in the night shift rotation at all (such as those who only work the day shift, administrative shift, and have no night shift duty responsibilities).

### 2.3. Measures

#### 2.3.1. Demographics

The general information questionnaire comprises 11 items, including gender, age, educational background, professional title, and monthly income.

#### 2.3.2. The Anxiety and Preoccupation About Sleep Questionnaire, APSQ

The APSQ was initially developed by Tang and Harvey [18] and subsequently revised by Jansson‐Frojmark et al. [19]. The Chinese version of the APSQ was translated and adapted by Shi Xuliang et al. [9]. This instrument is designed to assess sleep‐related concerns among night shift nurses. The scale comprises 10 items, each rated on a 5‐point Likert‐type scale ranging from 1 (“*strongly disagree*”) to 5 (“*strongly agree*”). The total score ranges from 10 to 50, with higher scores reflecting more pronounced sleep‐related concerns. In this study, the Cronbach’s *α* coefficient for the scale was 0.968, indicating high internal consistency.

#### 2.3.3. The Compassion Fatigue Short Scale, CF‐Short Scale

This scale was initially revised by Adams et al. [20], and the Chinese version was translated by the domestic scholar Lou Baona [21]. It is designed to measure the level of compassion fatigue in individuals. The scale comprises two dimensions—secondary trauma and job burnout—and includes a total of 13 items. A 10‐point Likert scale, ranging from 1 (“*never*”) to 10 (“*very frequently*”), is used, yielding a total score between 13 and 130. Higher scores indicate a greater level of empathy fatigue. In this study, the Cronbach’s *α* coefficient for the scale was 0.955, indicating high internal consistency.

#### 2.3.4. The Questionnaire for Nurse Intention to Remain Employed, QNIRE

This scale was originally developed by Turnley in 1998, and the Chinese version was translated by Tao Hong [22]. It is primarily used to assess employees’ intention to remain with an organization. The questionnaire comprises six items and employs a five‐point Likert‐type scale, with response options ranging from 1 to 5. The total score ranges from 6 to 30, with higher scores indicating a stronger intention to stay. The Cronbach’s alpha coefficient for this scale in the present study was 0.749.

### 2.4. Data Collection and Data Analysis

In this study, a convenience sampling method was employed to conduct a survey among 1831 on‐duty nurses from 8 tertiary general hospitals in China (located in Sichuan, Hubei, and Shenzhen). Prior to the commencement of the investigation, the research team underwent comprehensive training. Researchers meticulously screened potential participants based on predefined inclusion and exclusion criteria. The purpose of the study and instructions for completing the questionnaire were communicated to participants via WeChat. Upon obtaining informed consent from the respondents, the researchers administered the questionnaire online using SoJump (an online platform for collecting questionnaires: https://www.wjx.cn/). To ensure data accuracy and reliability, two researchers independently reviewed all completed questionnaires to assess their validity.

This study employed IBM SPSS 27.0 statistical software for data analysis. Measurement data were expressed as mean ± standard deviation (*M* ± SD), while count data were presented in terms of frequency and percentage. For data conforming to a normal distribution, intergroup comparisons were conducted using the independent samples *t*‐test or one‐way ANOVA, as appropriate. Pearson correlation analysis was performed to examine the relationships among key variables. Harman’s single‐factor test was applied to assess the potential presence of common method bias. Mediation effect analysis was carried out using the Hayes’ PROCESS macro in IBM SPSS 27.0. All statistical tests were conducted at a significance level of *α* = 0.05.

## 3. Results

### 3.1. Characteristics of Participants

Among the 1831 nurses surveyed, 1783 (97.4%) were female; 1308 (71.4%) had a bachelor’s degree or higher; 1348 (73.6%) were married; and 978 (53.4%) had a monthly income ranging from 5000 to less than 10,000 RMB. Other general demographic details are presented in Table 1.

**TABLE 1:** Personal characteristics and univariate analysis of retention intention (*N* = 1831).

Variable	Number (%)	The score of APSQ	The score of CF‐short scale	The score of QNIRE
Gender				
Female	1783 (97.4)	30.34 ± 11.32	47.80 ± 27.17	22.83 ± 3.68
Male	48 (2.6)	32.69 ± 9.55	56.67 ± 29.42	21.25 ± 4.38
*t*		−1.425	−2.226	2.923
*p*		> 0.05	< 0.05	< 0.05
Age				
18∼< 26	210 (11.5)	32.21 ± 10.88	50.78 ± 28.02	21.70 ± 3.60
26∼< 31	601 (32.8)	31.43 ± 11.47	48.05 ± 28.00	22.33 ± 3.79
31∼< 36	500 (27.3)	30.11 ± 11.15	48.37 ± 26.83	22.71 ± 3.56
36∼< 41	259 (14.1)	29.05 ± 11.11	48.63 ± 27.02	23.21 ± 3.59
41∼< 46	124 (6.8)	29.53 ± 11.63	47.37 ± 26.20	24.18 ± 3.32
46∼< 60	137 (7.5)	27.47 ± 10.72	41.99 ± 25.11	24.72 ± 3.48
*F*		4.935	1.834	17.888
*p*		< 0.05	> 0.05	< 0.05
Marital status				
Married	1348 (73.6)	29.80 ± 11.31	47.45 ± 27.17	23.06 ± 3.70
Unmarried	410 (22.4)	32.27 ± 10.95	50.70 ± 27.63	21.81 ± 3.60
Divorced, widowed, or other	73 (4.0)	30.92 ± 11.62	43.92 ± 26.11	23.37 ± 3.45
*F*		7.694	3.114	19.000
*p*		< 0.05	> 0.05	< 0.05
Educational background				
Junior college	523 (28.6)	30.79 ± 11.10	47.72 ± 27.72	22.73 ± 3.71
Bachelor’s degree or above	1308 (71.4)	30.24 ± 11.36	48.16 ± 27.09	22.82 ± 3.71
*t*		0.945	−0.306	−0.465
*p*		> 0.05	> 0.05	> 0.05
Professional title				
Nurse	267 (14.6)	31.16 ± 11.49	47.56 ± 28.97	22.21 ± 3.63
Nurse practitioner	738 (40.3)	31.04 ± 11.24	48.06 ± 27.48	22.41 ± 3.67
Nurse‐in‐charge	701 (38.3)	29.72 ± 11.23	48.41 ± 26.46	23.15 ± 3.72
Deputy chief nurse or above	125 (6.8)	28.78 ± 11.16	46.76 ± 26.90	24.28 ± 3.48
*F*		2.928	0.163	14.05
*p*		< 0.05	> 0.05	< 0.05
Department				
Internal medicine	688 (37.6)	31.01 ± 11.43	50.78 ± 28.20	22.54 ± 3.80
Surgery	432 (23.6)	30.68 ± 11.22	48.37 ± 27.25	22.75 ± 3.71
Pediatrics	86 (4.7)	29.16 ± 11.21	41.78 ± 24.59	23.34 ± 3.12
Obstetrics and gynecology	115 (6.3)	29.77 ± 11.28	45.53 ± 28.27	23.33 ± 3.19
Emergency department	83 (4.5)	28.45 ± 11.61	43.82 ± 26.95	23.43 ± 3.79
Other departments	87 (4.8)	29.45 ± 11.28	46.59 ± 26.54	22.55 ± 3.72
*F*		1.192	2.834	1.808
*p*		> 0.05	< 0.05	> 0.05
Years of work experience				
0∼< 4	212 (11.6)	30.96 ± 11.28	48.67 ± 27.36	21.77 ± 3.66
4∼< 6	319 (17.4)	32.30 ± 11.26	47.24 ± 27.95	22.38 ± 3.67
6∼< 11	426 (23.3)	31.00 ± 10.98	50.51 ± 27.20	22.23 ± 3.75
11∼< 15	521 (28.5)	30.11 ± 11.35	48.28 ± 27.27	23.04 ± 3.62
≥ 15	353 (19.3)	28.04 ± 11.22	45.01 ± 26.46	24.08 ± 3.47
*F*		6.737	2.081	19.510
*p*		< 0.05	> 0.05	< 0.05
Monthly income (RMB/month)				
0∼< 3000	106 (5.8)	33.30 ± 10.92	58.01 ± 31.36	21.32 ± 4.01
3000∼< 5000	699 (38.2)	30.13 ± 11.77	46.16 ± 27.83	22.64 ± 3.66
5000∼< 10,000	978 (53.4)	30.43 ± 10.91	48.52 ± 26.24	22.98 ± 3.69
≥ 10,000	48 (2.6)	27.33 ± 11.53	43.35 ± 24.84	24.44 ± 2.87
*F*		3.674	6.47	10.083
*p*		< 0.05	< 0.05	< 0.05
Frequency of night shifts per month				
0∼< 1	406 (22.2)	28.64 ± 11.55	45.59 ± 27.99	23.66 ± 3.63
1∼< 4	569 (31.1)	29.50 ± 11.12	47.94 ± 26.60	23.02 ± 3.58
4∼< 8	455 (24.8)	30.84 ± 10.82	46.73 ± 26.43	22.51 ± 3.66
≥ 8	401 (21.9)	32.96 ± 11.30	52.11 ± 28.02	21.90 ± 3.79
*F*		11.796	4.451	17.287
*p*		< 0.05	< 0.05	< 0.05
Weekly working hours				
0∼< 40	195 (10.6)	30.66 ± 11.62	46.80 ± 29.96	23.59 ± 3.32
40∼< 46	1038 (56.7)	29.69 ± 11.04	45.64 ± 25.83	23.03 ± 3.60
46∼< 50	402 (22.0)	31.15 ± 11.30	51.22 ± 27.29	22.43 ± 3.94
≥ 50	196 (10.7)	32.32 ± 11.93	55.42 ± 29.91	21.46 ± 3.75
*F*		3.889	9.568	14.568
*p*		< 0.05	< 0.05	< 0.05
Major traumatic events experienced in the past year				
No	1703 (93.0)	30.10 ± 11.23	47.16 ± 26.71	22.85 ± 3.70
Yes	128 (7.0)	34.37 ± 11.27	59.63 ± 31.66	21.93 ± 3.78
*t*		−4.134	−4.340	2.728
*p*		< 0.05	< 0.05	< 0.05

### 3.2. A Univariate Analysis of Sleep‐Related Worry, Compassion Fatigue, and Retention Intention Among Nurses With Varying Characteristics

The results of the univariate analysis indicated statistically significant differences in retention intention (QNIRE) scores among nurses with respect to gender, age, marital status, professional title, years of work experience, monthly income, frequency of night shifts per month, weekly working hours, and whether they had experienced major traumatic events within the past year (*p* < 0.05). Additional findings are presented in Table 1.

### 3.3. The Scores of Nurses’ Sleep‐Related Worry, Compassion Fatigue, and Retention Intention

The results of this study indicated that the mean sleep‐related worry score was 30.40 ± 11.28; the compassion fatigue score was 48.03 ± 27.26; and the retention intention score was 22.79 ± 3.71 (Table 2).

**TABLE 2:** Descriptive analyses of main study variables (*N* = 1831).

Variable	The quantity of items	Min	Max	Score ($\overset{¯}{x}$ * ± s*)
Sleep‐related worry	10	10	50	30.40 ± 11.28
Compassion fatigue	13	13	130	48.03 ± 27.26
Retention intention	6	6	30	22.79 ± 3.71

### 3.4. Correlation Analysis of Nurses’ Sleep‐Related Worry, Compassion Fatigue, and Retention Intention

The results of this study indicate that there is a negative correlation between nurses’ sleep‐related worry and their retention intention (*r* = −0.311, *p* < 0.01). The compassion fatigue of nurses is also negatively correlated with the retention intention (*r* = −0.411, *p* < 0.01). In addition, a positive correlation was observed between sleep worry and compassion fatigue (*r* = 0.585, *p* < 0.01). Refer to Table 3 for details.

**TABLE 3:** Correlation analysis of nurses’ sleep‐related worry, compassion fatigue, and retention intention (*N* = 1831).

Variable	Sleep‐related worry	Compassion fatigue	Retention intention
Sleep‐related worry	1	—	—
Compassion fatigue	0.585^∗∗^	1	—
Retention intention	−0.311^∗∗^	−0.411^∗∗^	1

^∗∗^ *p* < 0.01.

### 3.5. Analysis of the Mediating Role of Nurses’ Compassion Fatigue in the Relationship Between Sleep‐Related Worry and Retention Intention

**FIGURE 1:**
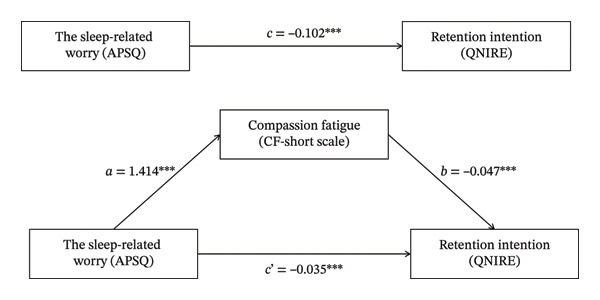
The mediating effect path of nurse compassion fatigue between sleep‐related worry and retention intention (*N* = 1831).

This study examined the mediating role of nurses’ compassion fatigue in the relationship between sleep‐related worry and retention intention. Several control variables were included in the analysis, such as gender, age, marital status, professional title, years of work experience, monthly income, number of night shifts per month, weekly working hours, and whether the individual had experienced a major traumatic event in the past year. The results demonstrated that sleep‐related worry significantly predicted compassion fatigue (*a* = 1.414, SE = 0.046, *p* < 0.001). Furthermore, compassion fatigue was found to significantly predict retention intention (*b* = −0.047, SE = 0.004, *p* < 0.001). Sleep‐related worry also showed a direct negative effect on retention intention (c’ = −0.035, SE = 0.009, *p* < 0.001). Bootstrap analysis with bias‐corrected percentiles revealed that the 95% confidence interval did not include zero, confirming that compassion fatigue partially mediated the association between sleep‐related worry and retention intention. This mediating effect accounted for 65.15% (=*a*×*b* ÷ c) of the total effect. Please refer to Table 4 and Figure 1 for further details.

**TABLE 4:** Analysis of the mediating role of compassion fatigue in the relationship between sleep‐related worry and retention intention (*N* = 1831).

Effect	Path	*β*	SE	95% CI	*t*	*p*
Direct effect	The sleep‐related worry (APSQ)⟶ Retention intention (QNIRE)	−0.035 (*c*’)	0.009	−0.052, −0.018	−4.082	< 0.001

Indirect effect	The sleep‐related worry (APSQ)⟶Compassion fatigue (CF‐Short Scale)	1.414 (*a*)	0.046	1.324,1.504	30.871	< 0.001
	Compassion fatigue (CF‐Short Scale) ⟶ Retention intention (QNIRE)	−0.047 (*b*)	0.004	−0.054,‐0.041	−13.332	< 0.001

Total effect	The sleep‐related worry (APSQ)⟶ Retention intention (QNIRE)	−0.102 (*c*)	0.007	−0.117, −0.088	−13.997	< 0.001

## 4. Discussion

### 4.1. The Current Status of Nurses’ Sleep‐Related Worry, Compassion Fatigue, and Retention Intention

The results of this study suggest that sleep‐related worry among nurses is at a moderate level, which aligns with findings from prior research in the field [23]. This phenomenon may be attributed to the unique characteristics of the nursing profession, including shift work systems, high workloads, and considerable psychological stress. These factors contribute to generally poor sleep quality among nurses, frequently manifesting as difficulties in initiating sleep, maintaining sleep, and experiencing early morning awakenings [24]. Sleep‐related worry not only contributes to heightened fatigue, impaired concentration, and diminished memory among nurses [25], thereby adversely affecting their work performance and the quality of patient care, but also increases the likelihood of various physical and mental health issues, such as cardiovascular diseases and depression, ultimately exerting a negative influence on nurse–patient relationships [26, 27]. This study also shows that the lower the nurse’s position and the more night shifts they have, and if they have experienced trauma within the past year, all these factors will affect the level of sleep‐related concerns of the nurses. Therefore, it is suggested that hospital administrators systematically plan the working hours and shift scheduling of nurses, with a focus on controlling the frequency of night shifts and avoiding consecutive night shifts, so as to effectively alleviate the professional pressure on the nursing staff.

The findings of this study suggest that the level of compassion fatigue among nurses is moderate, a result that aligns with those of previous related studies [16]. The results of this study show that nurses who have worked more night shifts per month and who have experienced trauma in the past year are more likely to suffer from compassion fatigue. This may be attributed to their prolonged exposure to high‐intensity and high‐pressure work environments, as well as their frequent interaction with patients’ suffering and negative emotions, which collectively increase the likelihood of experiencing symptoms associated with compassion fatigue, including emotional exhaustion, depersonalization, and a diminished sense of personal accomplishment [28]. It is important to recognize that compassion fatigue not only compromises the physical and mental well‐being of nurses, contributing to various psychological issues such as job burnout, but also adversely affects the quality of patient care, which may result in decreased patient satisfaction and an increased risk of medical errors [29, 30]. Therefore, it is of great practical significance for nursing managers to closely monitor and promptly intervene in the issue of nurses’ compassion fatigue. Specific actionable measures include: conducting compassion fatigue screenings using professional scales (such as ProQOL) every quarter, establishing psychological files for high‐risk nurses; setting up in‐hospital psychological counseling services, providing at least two half‐days of face‐to‐face counseling and a night psychological hotline each week; organizing one Mindfulness Stress Reduction or Balint Group activity per month, each lasting 60–90 min; and optimizing the shift scheduling system to ensure a 48‐h recovery period after night shifts and half‐day sleep protection time the next day. Through these systematic and regular measures, compassion fatigue among nurses can be effectively prevented and alleviated.

The findings of this study suggest that nurses’ retention intention is at a moderate level, a result that aligns with those of previous studies [6]. This phenomenon can be attributed to a range of contributing factors, such as excessive workloads, frequent night shifts, irregular work schedules, and occupational burnout. The cumulative impact of these elements increases the likelihood of nurses considering resignation or role transitions, consequently diminishing their retention intention [31]. In this study, it was found that nurses who are older, have more work experience, hold higher titles, have fewer night shifts, and have not experienced any traumatic events in the past year tend to have a higher retention intention. Conversely, the retention intention is lower among those lacking these characteristics. Based on the above research findings, it is recommended that hospital administrators implement a series of targeted measures. Regarding shift scheduling, a scientific, reasonable, and fair arrangement should be made to reduce the frequency of night shifts and ensure that nursing staff have sufficient recovery time. At the same time, positive mental health support programs should be introduced to help nurses effectively manage occupational stress. Moreover, organizational support should be strengthened to enhance employees’ sense of belonging, thereby increasing the retention rate of nurses.

### 4.2. The Relationship Between Nurses’ Sleep‐Related Worry, Compassion Fatigue, and Retention Intention

The findings of this study not only highlight the substantial prevalence of sleep‐related worry and compassion fatigue among hospital nurses but also elucidate the combined mechanism through which these factors influence nurses’ intention to remain in their positions. The findings of this study indicate that sleep‐related worry among nurses is positively associated with compassion fatigue and negatively correlated with their retention intention. Sleep disturbances exert their influence on the physiological level through two primary mechanisms. First, they may disrupt the normal functioning of the hypothalamic‐pituitary‐adrenal (HPA) axis, resulting in elevated cortisol levels and increased emotional reactivity. Second, sleep disturbances can impair prefrontal cortex function, which leads to a reduced capacity for executive control and emotional regulation [32]. Neuroimaging‐based research has demonstrated that among nurses with sleep efficiency below 80%, a distinct neural response pattern emerges when they are exposed to patients’ expressions of pain. This pattern is marked by heightened amygdala activation and diminished anterior insula activity. Specifically, the increased activation of the amygdala intensifies the perception of patients’ pain, whereas the reduced activation of the anterior insula impairs empathetic accuracy [33]. It is worth noting that such neural‐level changes are particularly pronounced among night shift nurses. Research data indicate that night shift work further compromises the sleep quality of nurses, thereby exacerbating empathy fatigue within this professional group by inducing emotional distress [34].

The negative predictive impact of sleep‐related worry on nurses’ retention intention is more pronounced. Specifically, fragmented sleep may reduce working memory capacity, consequently impairing an individual’s attention and judgment accuracy [32]. This can hinder nurses’ ability to manage complex medical instructions or respond effectively to unexpected situations, thereby contributing to a heightened sense of professional inadequacy. Simultaneously, sleep disturbances can compromise emotional regulation, increasing the likelihood of experiencing professional frustration [35]. Furthermore, insufficient sleep may lead to chronic fatigue and a negative attribution bias, wherein routine work challenges are perceived as systemic issues. This perception intensifies the desire to leave the profession, ultimately diminishing job retention [13].

The findings of this study suggest that compassion fatigue among nurses is negatively associated with their intention to remain in their current positions, a result that aligns with previous research in this area [36]. Research conducted by Wu et al. [37] indicates that when nurses’ compassion fatigue total score exceeds 65 points, their likelihood of leaving the profession increases by 3.2 times (OR = 3.2, *p* < 0.01). Among the various dimensions examined, job burnout demonstrates the strongest predictive power (*β* = 0.53). These findings suggest that emotional resource depletion, primarily manifested as compassion fatigue, can further lead to job burnout among nurses, potentially triggering occupational avoidance behaviors and ultimately diminishing their intention to remain in their current positions.

The research findings suggest that compassion fatigue partially mediates the relationship between nurses’ sleep‐related worry and their intention to remain in their current positions. Specifically, 65.15% of the effect of sleep‐related worry on retention intention is transmitted through the mediating mechanism of compassion fatigue. This result suggests that the sleep‐related worry experienced by nurses not only directly impacts their job stability but may also indirectly diminish their intention to remain in the profession by exacerbating the depletion of emotional resources, such as compassion fatigue. Specifically, the emotional stress and psychological burden resulting from sleep‐related worry can further intensify compassion fatigue among nurses, thereby negatively influencing their intention to remain in their positions. This finding further supports Hypothesis 3, which was formulated based on the theory of resource conservation. Specifically, it indicates that compassion fatigue mediates the relationship between nurses’ sleep‐related worry and their intention to remain in their current positions. Sleep, as a crucial personal resource, plays a vital role in maintaining psychological well‐being; therefore, a decline in its quality can directly result in a depletion of psychological resource reserves [38]. When nurses are exposed to emotionally demanding work scenarios, such as hospice care and trauma care, their remaining psychological resources may become insufficient to sustain a high level of empathetic engagement. As a result, a resource conservation mechanism is activated, leading to manifestations of compassion fatigue, including emotional detachment and professional burnout [39, 40]. Ultimately, when resources continue to be depleted without sufficient replenishment, nurses may develop the intention to leave as a means of preventing further resource loss.

Based on the above research findings, it is recommended that hospital management give priority to addressing the concerns of nurses’ sleep‐related worry and compassion fatigue. These factors have been proven to be the key risk factors that undermine nurses’ retention intention. Specifically, excessive frequency of night shifts and excessive working hours per week will severely compress the physiological recovery period of nurses, exacerbate sleep deprivation and cumulative fatigue, and thereby cause them to have a retreat mentality toward their profession; nurses who have experienced major traumatic events (such as workplace violence, etc.) within the past year have significantly higher levels of compassion fatigue and are more likely to experience emotional exhaustion and a tendency to leave the profession. Therefore, a variety of intervention strategies should be implemented: adopt evidence‐based shift scheduling, control the frequency of night shifts to 4–6 times per month, avoid consecutive night shifts, and ensure a 48‐h recovery period; establish a psychological counseling mechanism within 72 h after a traumatic event; conduct sleep hygiene education and short‐term cognitive behavioral intervention; and regularly organize mindfulness reduction and empathy balance training. These measures can effectively alleviate sleep problems and compassion fatigue, ultimately enhancing nurses’ retention intention.

### 4.3. Limitations

This study has a certain value, but it still has limitations. Firstly, due to the cross‐sectional design, all variables were measured at the same point in time, which prevented us from confirming the causal direction of the mediating role of compassion fatigue between sleep‐related worry and retention intention. Specifically, although the statistical model supported the good fit of compassion fatigue as the mediating path, the cross‐sectional data could not rule out the possibility of reverse causality or the influence of omitted variables. Therefore, the presented mediating effect in the report should be regarded as an exploratory association rather than a causal inference. Future studies should use longitudinal tracking or intervention research to verify the temporal sequence and causal mechanism among the variables. Secondly, although the samples were collected from eight hospitals, due to the completely anonymous nature of the survey and the absence of hospital identification codes, we were unable to count the specific number of participants from each hospital, nor could we control for the clustering effect at the hospital level (such as the influence of departmental working environment and management style on the results). This limitation directly leads to two consequences: Firstly, it is impossible to assess the degree to which hospital heterogeneity interferes with the main results, which may weaken the robustness of the conclusion; secondly, the representativeness of the sample and potential selection bias are difficult to quantify (e.g., in some hospitals, nurses who pay more attention to sleep problems may be more inclined to participate), thereby limiting the generalization ability of the results to other medical institutions. To address this deficiency, we suggest that future research allocate unique codes to each hospital during the design stage, incorporate multilevel models while ensuring anonymity, or report the sample size and response rate of each center.

## 5. Conclusion

In this multicenter cross‐sectional study, we explored the mediating role of nurse compassion fatigue in the relationship between sleep‐related worry and retention intention. The results showed that nurses were at a moderate level in terms of sleep‐related worry, compassion fatigue, and retention intention. This highlights the importance of addressing sleep issues and compassion fatigue to support nurses’ career development. The study emphasizes that healthcare institution managers should adopt comprehensive strategies to alleviate nurses’ sleep‐related worry and compassion fatigue, and enhance their sense of professional value. Such measures not only help to increase nurses’ work engagement but also provide valuable empirical evidence for future human resource management and career development, ultimately effectively reducing the turnover rate of nurses.

## 6. Implications for Nursing Management

This study reveals the complex mechanism by which nurses’ sleep‐related worry affects their retention intention through compassion fatigue and clearly points out the crucial mediating role of compassion fatigue in this process. Specifically, sleep‐related worry not only may weaken nurses’ physical and mental recovery capabilities but also may exacerbate the physical and mental exhaustion and empathy pressure they accumulate during their care of patients. Ultimately, this could further exacerbate the level of compassion fatigue among nurses, indirectly altering their retention intention and creating a vicious cycle. This finding provides important practical guidance for nursing managers and policymakers: To break this chain, it is not enough to solely focus on sleep problems or emotional stress; instead, it is necessary to intervene simultaneously at the system level on both sleep health and compassion fatigue. This can be achieved by implementing targeted management strategies, such as optimizing the shift scheduling system to ensure nurses’ continuous sleep time, establishing quiet rest spaces to alleviate sleep anxiety, regularly conducting Balint groups or mindfulness training to relieve empathetic stress, and establishing psychological support networks to reduce the risk of emotional exhaustion. Therefore, it is recommended that nursing managers incorporate “sleep‐friendly working environment” and “compassion fatigue prevention mechanism” into their daily management standards and collaborate with hospital policymakers to ensure nurses’ rest rights and psychological support resources at the institutional level. This may change the retention intentions of nurses and alleviate the problem of talent loss.

## Author Contributions

Feng Peng, Xiaoying Zeng, and Jue Wu conducted the research design and collected data. Feng Peng and Jue Wu analyzed the data and wrote the manuscript. Feng Peng, Xiaoying Zeng, and Jue Wu revised the manuscript. Feng Peng and Xiaoying Zeng contributed equally to this work.

## Funding

This research was supported by the Sichuan Medical and Health Care Promotion Institute Scientific Research Project (Grant No. KY2024SJ0232) and the Primary Health Development Research Center of Sichuan Province Program (Grant No. SWFZ24‐Y‐21).

## Disclosure

All authors approved the final version for submission.

## Ethics Statement

This study was examined and formally approved by the Ethics Committee of Sichuan Taikang Hospital (No. SCTK‐IRB‐2024–014). All data collection was conducted in accordance with the principle of informed consent. Participants retained the right to withdraw from the study at any point during the research process. Furthermore, strict confidentiality was maintained throughout the data collection procedure, and we solemnly affirm that the collected data will be used exclusively for statistical analysis purposes.

## Conflicts of Interest

The authors declare no conflicts of interest.

## Data Availability

The raw data supporting the conclusions of this article will be made available by the authors without undue reservation.
